# Exercise Prescription in Lung-Transplanted Cystic Fibrosis Adults

**DOI:** 10.3390/jfmk10020212

**Published:** 2025-06-04

**Authors:** Melissa Orlandi, Maria Stella Pinnarò, Marco Corsi, Beatrice Borchi, Annalisa Cavallo, Sandra Guarducci, Alessandro Bartoloni, Martina Donati, Cecilia Defraia, Leonardo Nesi, Stefano Sparacio, Claudia Mannini, Federico Lavorini, Vittorio Bini, Silvia Bresci, Laura Stefani

**Affiliations:** 1 Sports Medicine Centre, University of Florence, 50100 Florence, Italy; orlandime@aou-careggi.toscana.it (M.O.); mariastella.pinnaro@gmail.com (M.S.P.); marco.corsi@unifi.it (M.C.); 2 Cystic Fibrosis Centre, AOUC, 50100 Florence, Italy; borchib@aou-careggi.toscana.it (B.B.); cavalloa@aou-careggi.toscana.it (A.C.); guarduccis@aou-careggi.toscana.it (S.G.); alessandro.bartoloni@unifi.it (A.B.); brescis@aou-careggi.toscana.it (S.B.); 3 Rehabilitation Service, AOUC, 50100 Florence, Italy; donatima@aou-careggi.toscana.it (M.D.); defraiac@aou-careggi.toscana.it (C.D.); nesil@aou-careggi.toscana.it (L.N.); sparacios@aou-careggi.toscana.it (S.S.); 4 Pneumology and Respiratory Physiology, AOU Careggi, 50100 Florence, Italy; manninic@aou-careggi.toscana.it (C.M.); federico.lavorini@unifi.it (F.L.); 5 Medicine Department, University of Perugia, 06100 Perugia, Italy; binivittorio@gmail.com

**Keywords:** exercise prescription, cystic fibrosis, transplantation

## Abstract

**Background**: Physical exercise intervention in cystic fibrosis (CF) is of recent interest; however, no specific method to detect improvements in body composition and cardiovascular performance after transplantation has been investigated. This study aims to verify the feasibility of an exercise prescription program in CF lung-transplanted patients compared to other solid organ transplanted recipients (OLT) in terms of cardio-respiratory and body composition performance. **Methods**: The two groups, trained with a moderate intensity program, were evaluated by body composition analysis and a cardiopulmonary test (CPET) and compared to healthy subjects (HS). **Results**: A total of 10 CF, 10 OLT, and 10 HS were included. BMI was significantly lower in the CF group with lower total and appendicular free fat mass (*p* = 0.01). The CF group also showed significantly lower functional and cardiovascular parameters in the CPET test (peak VO2, VOR/HR) compared to the OLT and HS groups, but similar ventilatory response (VE/VCO2 slope) to OLT. In the CF group, free fat mass and functional parameters (peak VO2 and VO2/HR) were negatively correlated (r = −0.51 and −0.52, respectively). **Conclusions**: CF patients would benefit from an individualized exercise prescription program to improve all cardiovascular parameters, overall body composition, and, consequently, related respiratory parameters. Peak VO2 and body composition should be largely used to plan exercise prescription program among transplanted CF.

## 1. Introduction

Cystic fibrosis (CF) is a complex disease that requires multidisciplinary management [1,2,3,4] with potential limitations in lifestyle behaviors and physical activity before accurate therapy and organ transplantation. The physical activity regimen during the post-transplantation phase in this specific category has not been thoroughly investigated, despite the fact that the role of individualized exercise has been previously evaluated in many chronic metabolic diseases [5,6,7,8,9], as well as in individuals with solid organ transplants [10,11,12,13,14]. Specific guidelines for transplant recipients are currently lacking, although the post-transplant condition has been explored in terms of mixed physical activity interventions. Organ transplantation, however, represents an area of interest for the application of therapeutic strategies involving physical exercise for managing disease complications, particularly in the supervision of respiratory failure and the reduction in cardiovascular risk factors. The fragility caused by the underlying disease, as in the case of CF, is further compounded by the challenges of post-transplant management. No specific experiences have been reported regarding physical activity in CF, especially in the case of bilateral lung transplantation. Previous studies have shown that the correct optimization of the exercise prescription also depends on the assessment of body composition, including estimates of nutritional and hydration status through bioelectrical impedance analysis [12,14,15]. This study primarily aims to assess the feasibility of a tailored exercise program for CF patients with bilateral lung transplantation, evaluated by a cardiopulmonary exercise test. Additionally, it seeks to provide a detailed analysis of how training levels can influence body composition, with a particular focus on muscle distribution and water balance in various anatomical regions.

## 2. Materials and Methods

A total of 10 double-lung transplant patients due to cystic fibrosis (CF: 8 men and 2 women, mean age 34 ± 7.45 years) who accessed the exercise prescription program for the first time at the Sports Medicine clinic of the University Hospital of Florence were included in this study. Patients were referred to the Sports Medicine Unit as part of a routine clinical protocol aimed at evaluating long-term transplant outcomes, including sarcopenia screening. This observational study was part of a prospective evaluation of exercise capacity and body composition in clinically stable patients. The investigation took place from November 2024 to February 2025. The inclusion criteria were being at least one-year post-transplant (mean time to transplantation: 6.54 ± 1.64 years) and clinically stable (e.g., absence of lung-related complications in the previous six months, including acute rejection episodes and reduced spirometry parameters).

This group was compared to two other groups. The first control group consisted of 10 liver transplant patients (OLT: 9 men and 1 woman, mean age 57.7 ± 4.34 years, and mean time to transplantation: 8.1 ± 2.87 years), who had been attending the exercise prescription program at the Sports Medicine clinic of the University Hospital of Florence for at least one year. All OLT patients were clinically stable, with no liver-related complications in the previous six months, including acute rejection episodes or increased serum transaminases above twice the upper limit of normal. The second control group consisted of 10 healthy active subjects (HS: 6 men and 4 women, mean age 38.9 ± 11.62 years).

All participants provided informed consent, and the same tests were conducted for each subject throughout this study.

The exclusion criteria included combined transplantation, re-transplantation, physical limitations, cardiovascular contraindications to exercise, and psychiatric or severe debilitating neurological disorders. All participants in the two transplanted groups were on immunosuppressive therapy, including calcineurin inhibitors (Ciclosporin or Tacrolimus) in combination with Mycophenolate or Everolimus, and steroids (Methylprednisolone). Moreover, 90% of the CF subjects were on Ciclosporin and Everolimus, while the remaining 10% was on Tacrolimus and Everolimus. Additionally, 65% of OLT were on Tacrolimus and Everolimus; the remaining 45% was on Ciclosporin with Everolimus or Mycophenolate. All patients were on steroid treatment. None of the participants had major arrhythmias or cardiac events in the months leading up to this study.

The two transplanted groups underwent a physical exercise program consisting of a mixed physical activity regimen (including aerobic and resistance training) at least three times a week for 30 min at an intensity of approximately 60% of their maximum heart rate in an unsupervised manner. The Karvonen formula, based on the heart rate range of the two thresholds, was used to individually determine the heart rate range for aerobic activity [16]. The International Physical Activity Questionnaire (IPAQ) was used to measure the adherence to the physical activity program [17], considering <700 Mets/k/week as sedentary behavior, 700 to 2200 Mets/k/week as compatible with moderate activity levels, and values >2200 Mets/k/week as very active.

Nutritional habits were evaluated by MEDI-LITE adherence score, consisting of nine items that assess the daily consumption of fruit, vegetables, cereals, meat and meat products, dairy products, alcohol, and olive oil, and the weekly consumption of legumes and fish. The final score ranges from 0 (low adherence) to 18 (high adherence to the Mediterranean diet) [18].

After a clinical and anamnestic evaluation, all the participants underwent a body composition evaluation by measuring nutritional and hydration status in a resting condition and a functional evaluation by a cardiopulmonary exercise test (CPET).

### 2.1. Bioimpedance Measurement and Body Composition Assessment

Bioimpedance analysis (BIA) is a safe, fast, non-invasive, and cost-effective technique used to estimate body composition in clinical practice and population studies [19]. The BIA works on the principle of passing a low-intensity alternating electric current (approximately 800 μA) at 50 kHz through the human body, which travels at different speeds depending on the body’s composition. Tissues with a higher percentage of “lean” mass, mainly composed of water and ions, facilitate the passage of this current, in contrast to “fat” tissues, which are less rich in water. Lean tissues, such as bone and muscle, have a more hydrated cellular population than adipocytes in adipose tissue, allowing BIA to estimate body composition with reasonable accuracy. One of the main parameters measured by this technique is impedance (Z), determined by resistance (R), which represents the ability of biological structures to resist the flow of current, and reactance (Xc), indicating the force opposing current passage due to cellular membranes and cellular mass (BCM). Another related value is the phase angle (PhA), which describes the relationship between resistance (R) and reactance (Xc). This parameter depends on the subject’s hydration status (and the extracellular/intracellular ratio) and their cellular mass (BCM), offering insights into membrane integrity and cellular functionality.

An Akern BIA 101 BIVA^®^ PRO bioimpedance analyzer (Pisa, Italy) was used in this study. The standard procedure required the patient to lie in a supine position, at rest for a few seconds, on a flat surface, free of any metal objects, with their arms and legs spread apart at 30° and 45°, respectively. After resting, the skin on the right hand and right foot was cleaned, and two electrodes were placed on the back of the corresponding hand and foot. The following values were measured before the total BIA analysis in order to assess body composition: weight (kg) and height (m) were measured using a mechanical scale with a stadiometer in an upright position, without shoes, with an approximation to the nearest 0.1 (kg or m) for excess or deficit. Body Mass Index (BMI) (kg/m^2^) was calculated as the ratio of weight (kg) to height squared (m^2^). For body composition, the following parameters were considered: lean mass (FFM), fat mass (FM), appendicular lean mass also calculated with Jansenn formula (J FFM) [20], cell mass (BCM), intracellular water (ICW), extracellular water (ECW), total body water (TBW) expressed as a percentage (%), phase angle (PhA) expressed in degrees (°), and the Hydragram hydration index, expressed as the ratio between TBW and FFM [20].

In addition to total BIA, regional BIA (Akern BIA 101 BIVA^®^ PRO, Pisa, Italy) was also conducted. This technique enables the obtainment of impedance (Z), resistance (R), reactance (Xc), and phase angle (PhA) for the ten analyzed body segments: right and left upper limbs, right and left lower limbs, superior segment (thorax and right and left arm), inferior segment (abdomen and right and left leg), right and left hemisome, and left and right thorax.

In order to obtain the 10 anatomical regions, 4 additional electrodes (2 on the back of the left hand and 2 on the back of the left foot) were added.

### 2.2. Cardiopulmonary Test (CPET)

The CPET was conducted according to established guidelines [21] using an electromagnetic brake cycle ergometer (Ergoline) and a specific gas measurement machine (COSMED Quark CPET, Albano Laziale, Rome, Italy). Each participant was instructed to avoid strenuous physical exertion the day before the test and to abstain from consuming solid foods or carbohydrate-rich drinks for three hours before the test. The test was performed in the morning under controlled conditions (temperature: 18–24 °C; humidity: 30–60%). The ramp protocol for cardiopulmonary testing was tailored based on gender and body composition to aim for muscle exhaustion between 8 and 12 min. Participants wore an oro-facial mask connected to a gas-measuring device. Exhaled CO_2_ and O_2_ consumption were measured breath by breath. The lowest possible increase in watts (1, 2, or 5 watts) was set for each ramp to achieve the most linear increase in load and, therefore, a more physiological response. After 3 min of warming up by cycling without load at 50 rpm, the test followed these steps: at the start of the actual effort, cycling was required at a cadence between 60 and 80 rpm until muscle exhaustion. The test finishes when participants could no longer maintain their cycling cadence despite verbal encouragement. The test was considered maximal if at least two of the following criteria were met: Respiratory Exchange Ratio (RER) > 1.10, maximum heart rate > 85% according to age, and a plateau in oxygen consumption (increase < 150 mL·min^−1^) in the last 30 s of the test. The test was stopped early in the presence of cardiovascular signs and symptoms (complex ventricular arrhythmias, drops in systolic blood pressure, dizziness, etc.). Continuous monitoring included a 12-lead ECG and oxygen saturation. During the test, various parameters were measured, including oxygen consumption (VO2), carbon dioxide production (VCO2), tidal volume (VT), respiratory rate (RR), minute ventilation (VE), heart rate (HR), and workload (WR). The lactate threshold was determined using the V-slope and ventilator equivalents approach. Other variables analyzed included the relationship between oxygen consumption and heart rate (VO2/HR, a measure of stroke volume), the relationship between oxygen consumption and workload (VO2/W slope, a measure of circulatory efficiency), the product of VO2 peak (mL/kg/min) and systolic blood pressure (a measure of circulatory strength) and the relationship between VCO2 and VE (VCO2/VE or VCO2 slope), a measure of ventilatory efficiency.

### 2.3. Informed Consent and Ethical Considerations

Informed consent was obtained from all subjects involved in this study. The local Independent Ethics Committee approved this study (CEAVC, Tuscany, Italy, approval number 20659) that was developed according to the ethical parameters established in the Declaration of Helsinki (1964) and its later amendments. This study is a part of a larger investigation in transplanted subjects. The local Independent Ethics Committee approved this study (study ID: ISRCTN66295470, Tuscany, Italy), which was developed according to the ethical parameters established in the Declaration of Helsinki (1964) and its later amendments [22]. The investigation has been also approved and conducted in a public hospital by a specific sanitary protocol (code 23OT20).

### 2.4. Statistical Analysis

Data are expressed as the mean and standard deviation; data analyzed were at baseline. Analysis was made using SPSS Statistics 30.0.0 Grad Pack 2024 software (IBM, Armonk, NY, USA). The group comparisons were analyzed using one-way ANOVA, followed by post hoc pairwise comparison and the statistically significant value was set at *p* < 0.05. A confidence interval (CI) was also determined. A Spearman correlation test was also performed between the mentioned data.

## 3. Results

Data are expressed as mean ± standard deviation (SD), and for some parameters, the confidence interval (CI) was also reported. CF patients showed a lower and statistically different age compared to OLT but not to HS (Table 1). BMI was significantly lower in CF compared to OLT (*p* = 0.03), particularly with lower values of FFM compared to the other two groups (*p* = 0.003; vs. OLT (*p* = 0.01) vs. HS (*p* = 0.02)). This is more evident if considering J-FFM: CF showed a significant reduction in J-FFM compared to the other two groups (*p* = 0.02), with mean values of 19.4 ± 3.6 Kg in CF (CI: 16.81–22.01), 23.4 ± 2.5 kg in OLT (CI: 21.58–23.44) (*p* = 0.05), and 23.3 ± 4.4 kg in HS (CI: 20.61–23.42) (*p* = 0.01). FM, BMC, and hydration parameters (ECW and ICW) were similar in all groups (Table 2). Preliminary regional BIA results were comparable and similar in the three populations (Table 3).

CPET showed significantly lower functional and cardiovascular parameters (peak VO2 and VO2/HR, *p* < 0.001) in CF compared to OLT (*p* = 0.003 and 0.002, respectively) and to HS (*p* < 0.001). The respiratory response (VE/VCO2) was normal in HS but similar and augmented in CF and OLT (*p* = 0.03) (Table 1). Reduced functional parameters of the two transplanted groups compared to HS were also confirmed by the different IPAQ results reported (*p* < 0.001), with mean lower values in CF and OLT. MEDI-LITE questionnaire showed a sufficient but not optimal adherence to the Mediterranean diet in all groups (Table 1).

**Figure 1:**
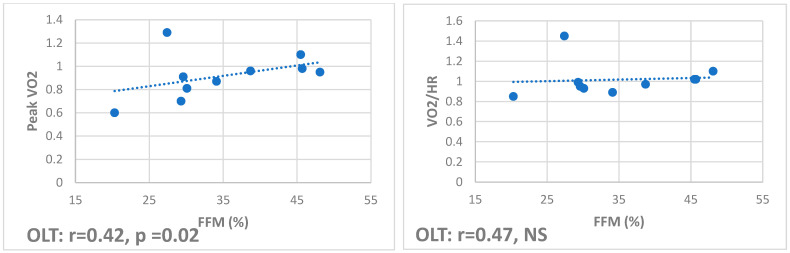
Correlations in OLT between free fat mass and peak VO2 and VO2/HR, respectively. This group showed a positive correlation between cardiovascular fitness and lean body mass.

**Figure 2:**
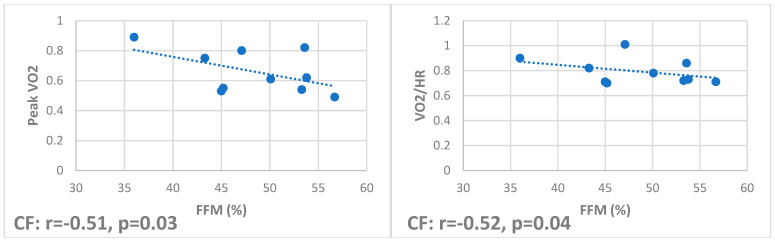
Correlations in CF between free fat mass and peak VO2 and VO2/HR, respectively. This group showed a negative correlation between cardiovascular fitness and lean body mass.

Correlations showed that total and appendicular FFM positively correlate with oxygen pulse (r 0.54, *p* = 0.01, and r 0.47, *p* < 0.01, respectively) and peak VO2 (r 0.47, *p* = 0.03, and r 0.41, *p* = 0.02, respectively) in HS but only with peak VO2 (r 0.42, *p* = 0.02, and r 0.2, *p* = 0.04, respectively) in OLT (Figure 1), while they negatively correlate in CF (r −0.51, *p* = 0.03, and r −0.52, *p* = 0.04, respectively) (Figure 2).

## 4. Discussion

Cystic fibrosis is a frail syndrome that could be included in the exercise prescription program at any stage of the disease also after transplantation. The lifestyle management and reconditioning program could represent an important aspect in terms of gaining quality of life. Lung transplantation represents a specific context in which the exercise adaptability can be compromised and therefore susceptible to improvement with regular physical exercise.

Few studies report experiences from the lung transplanted subjects [23,24], and, especially, the CF transplanted group, when the pulmonary district is involved, has not yet been investigated. Given the extreme fragility and complexity of this category, CF patients have historically been excluded from these types of programs due to the fear of contagion, considering the high intrinsic risk of infection in communitarian settings like gyms.

The results of this study have investigated this specific category regarding dietary habits and exercise prescription. The results emphasized some parallelism, and therefore similar behaviors of many parameters of the cardiopulmonary investigations, between CF patients and frail patients who have undergone other kinds of solid organ transplantation. Both groups, with and without CF, exhibited low levels of spontaneous physical activity associated with a reduction in muscle mass, particularly appendicular muscle mass. This is even more evident when considering appendicular muscle mass, whose deficiency defines sarcopenia: transplant patients often experience lean mass depletion due to sedentary behavior and the accumulation of cardiovascular risk factors. Given the well-known correlation between lean mass and cardiovascular fitness levels [14,25,26,27], physical exercise gains even greater relevance, as a reduction in VO2 max is associated with an increased risk of mortality. An important observation is that, in this specific category, a positive trend of the correlation between the FFM and the cardiovascular function parameters (VO2 max and VO2/HR) has been found. Given the reduced sample size, the results should be considered as preliminary.

Furthermore, CF patients showed a reduced aerobic capacity. Our data show that the main differences in functional parameters between CF and transplant patients are related to cardiovascular rather than respiratory parameters.

This suggests that the differences in fitness levels may be due to reduced training levels and cardiovascular function, contrary to what might be expected in patients with lung disease. Therefore, including these patients in exercise programs may have even greater benefits.

In contrast, regional BIA did not show significant differences between the two groups. This may be partly due to the small sample size, but it is clear that there is currently no significant compartmentalization of body composition in these groups, as confirmed by the absence of differences in water distribution as assessed by standard BIA.

It has been demonstrated that structured physical exercise provides benefits in the frail populations studied [13,14,28]. It would therefore be advisable to encourage such activities among CF patients, just as in transplant recipients, with the goal of increasing muscle strengthening.

The follow-up needs to include motivation to perform resistance exercises that can increase muscle mass and improve overall body composition in these patients.

Regarding dietary aspects, poor adherence to the Mediterranean diet was investigated using the MEDI-LITE questionnaire. Adherence to the Mediterranean diet should therefore be encouraged.

Among these groups, CF emerged as the condition most affected by sarcopenia, likely due to higher levels of chronic systemic inflammation from the underlying disease, despite patients often following high-fat diets. These patients are unable to increase muscle mass even with an unbalanced diet, a situation not observed in the control group. In transplant recipients, despite having higher absolute values of lean mass, body composition remains unbalanced, probably due to a lack of physical exercise in addition to poor dietary habits.

The questionnaires used to assess adherence to the Mediterranean diet administered to these two populations showed no significant differences, while standard BIA played a more important role in stratifying these categories. The questionnaires used, such as the IPAQ to assess physical activity levels and the MEDILITE to evaluate adherence to the Mediterranean diet, have shown that both of these frail categories tend to neglect both nutritional aspects and physical activity.

“Time to transplantation” is directly correlated to the cardiotoxicity of maintenance therapy, which leads to the secondary accumulation of additional risk factors beyond the underlying disease. For the most sarcopenic individuals, special attention has been given to the importance of physical exercise for therapeutic purposes—not only to improve both total and regional body composition but also to mitigate the cardiotoxic effects of chronic immunomodulatory therapy. Thus, the role of physical exercise should not only be considered as therapeutic but also primarily as preventive.

## 5. Conclusions

This study, although preliminary, highlights the importance of assessing cardiovascular performance and body composition by standard bioelectrical impedance analysis (BIA) and regional BIA in people with CF after transplantation. The substantial lack of differences between the two groups of transplanted subjects suggests the possibility to extend the exercise program to these subjects, without any substantial modifications.

Despite regional BIA being a useful and non-invasive method capable of providing detailed information on specific body areas—particularly for assessing compartments such as the lower limb segment—this particular case did not reveal any significant differences between the different populations. Further studies with a larger sample size may provide more conclusive results.

CF patients would benefit from an individualized exercise prescription program to improve all cardiovascular parameters, overall body composition, and consequently related respiratory parameters. The correct lifestyle is fundamental in those categories at potential risk for high levels of comorbidities, and the adherence to an individualized regimen of physical activity can be considered in a context of a therapeutical program for secondary prevention.

In conclusion, considering that CF subjects have not yet been considered for exercise prescription programs, the data obtained, although preliminary, are indicative of a potential inclusion of this category; these protocols are in fact already validated for other transplanted subjects.

At present, CF patients can represent a new category that could be considered in sport medicine for an individualized physical exercise prescription. The program proposed could be addressed toward a therapeutical approach in order to improve fitness, reduce cardiovascular risk, better manage the underlining condition, and prevent sarcopenia.

### Limits of the Study

This is the first investigation in this field, exploring the potential application of exercise prescription in CF. The restricted group of subjects studied and the missing data from the periodical follow-up create some difficulties for a large and correct interpretation of the results shown. Further studies will therefore be necessary to confirm this evidence.

## Figures and Tables

**Table 1:** Principal anthropometric and functional parameters.

	CF	OLT	HS	*p*	P (CF-OLT)	P (CF-HS)
Age	39.9 ± 11.62	57.7 ± 4.34	34 ± 7.45	<0.001	0.0001	NS
BMI	22.2 ± 2.15	24.9 ± 2.78	23.83 ± 2.96	NS	0.03	NS
TT (years)	6.54 ± 1.64	8.1 ± 2.87	None	NS	NS	NS
IPAQ	1264 ± 962.30	747 ± 548.47	2668 ± 1173.23	<0.001	NS	0.009
MEDI-LITE	11.7 ± 1.88	11.4 ± 1.89	12.6 ± 2.17	NS	NS	NS
Peak VO2 (%)	0.66 ± 0.14	0.91 ± 0.19	1.24 ± 0.23	<0.001	0.003	0.000
VO2/HR (%)	0.79 ± 0.10	1.01 ± 0.16	1.32 ± 0.22	<0.001	0.002	0.000
VE/VCO2	37.35 ± 5.71	34.24 ± 5.49	26.91 ± 2.58	0.03	0.23	0.0002

Caption: BMI: body mass index; TT: time to transplantation; IPAQ: International Physical Activity Level Questionnaire; MEDI-LITE: Mediterranean Diet Adhesion Questionnaire; Peak VO2 %: percentage of maximum oxygen consumption on peak predicted value for the subjects; VO2/HR %: percentage of peak oxygen pulse predicted value for the subjects; VE/VCO2: slope between ventilation and carbon dioxide production; NS: not significant.

**Table 2:** Principal standard BIA parameters.

	CF	OLT	HS	*p*	P (CF-OLT)	P (CF-HS)
PhA	6.2 ± 0.8	6.69 ± 1.4	6.4 ± 0.9	NS	NS	NS
FFM (Kg)	43.11 ± 16.02	58.29 ± 5.13	58.4 ± 8.52	0.003	0.01	0.02
FFM%	0.81 ± 0.0537	0.81 ± 0.06	0.80 ± 0.05	NS	NS	NS
FM (Kg)	11.74 ± 3.79	14.24 ± 5.96	14.4 ± 4.40	NS	NS	NS
FM%	0.261 ± 0.18	0.19 ± 0.05	0.19 ± 0.05	NS	NS	NS
ASMM (Kg)	19.41 ± 8.63	23.39 ± 2.53	23.33 ± 4.3	0.02	0.01	0.05
ASMM%	41.29 ± 4.73	39.18 ± 7.92	41.88 ± 5.0	NS	NS	NS
JFFM	25.55 ± 4.85	34.88 ± 9.25	30.57 ± 5.8	0.02	0.01	0.05
Rz	554.01 ± 83.28	475.21 ± 42.66	504.35 ± 63.67	0.04	0.02	NS
Xc	59.53 ± 11.23	48.76 ± 17.23	56.84 ± 6.43	NS	NS	NS
BM	1523.94 ± 120.22	1717.15 ± 152.26	1704.1 ± 189.6	0.02	0.006	0.02
BCM	0.52 ± 0.094	0.57 ± 0.06	0.55 ± 0.04	NS	NS	NS
ECW	0.45 ± 0.04	0.42 ± 0.06	0.43 ± 0.03	NS	NS	NS
ICW	0.54 ± 0.043	0.57 ± 0.06	0.57 ± 0.02	NS	NS	NS
TBW	0.59 ± 0.041	0.59 ± 0.04	0.59 ± 0.03	NS	NS	NS

Caption: PhA: phase angle; FFM: lean mass; FM: fat mass; ASMM: appendicular lean mass; J FFM: appendicular mass calculated with Jansenn formula; Rz: resistance; Xc: reactance; BM: basal metabolism; BCM: body cell mass; ECW: extracellular water; ICW: intracellular water; TBW: total body water. NS: not significant.

**Table 3:** Principal regional BIA parameters.

	CF	OLT	HS	*p*
Right hemisome				
Rz	536.3 ± 100.19	473.14 ± 44.53	510.81± 62.93	Ns
Xc	58.76 ± 11.05	52.82 ± 9.89	57.39 ± 6.24	Ns
PhA	6.02 ± 0.84	6.28 ± 1.11	6.43 ± 0.86	Ns
Left hemisome				
Rz	536.58 ± 107.17	481.74 ± 51.92	511.73± 61.69	Ns
Xc	53.08 ± 20.41	53.15 ± 8.68	56.99 ± 6.34	Ns
PhA	6.17 ± 0.8	6.22 ± 0.83	6.35 ± 0.78	Ns
Right upper limb				
Rx	255.14 ± 32.70	233.49 ± 23.77	246.81± 43.03	Ns
Xc	26.93 ± 3.65	26.75 ± 3.38	27.31 ± 2.59	Ns
PhA	5.84 ± 0.77	6.54 ± 1.25	6.45 ± 1.25	Ns
Left upper limb				
Rz	260.28 ± 52.46	236.63 ± 24.72	246.51± 44.32	Ns
Xc	26.08 ± 40.77	26.24 ± 4.28	27.09 ± 2.66	Ns
PhA	5.78 ± 0.454	6.4 ± 1.24	6.39 ± 1.15	Ns
Right lower limb				
Rz	257.51 ± 64.19	221.57 ± 38.11	245.81± 26.44	Ns
Xc	29.77 ± 6.07	25.58 ± 5.68	28.3 ± 3.59	Ns
PhA	6.63 ± 1.12	6.54 ± 1.18	6.55 ± 0.76	Ns
Left lower limb				
Rz	267.17 ± 70.22	203.82 ± 73.88	246.64± 27.95	Ns
Xc	29.61 ± 6.09	24.91 ± 5.70	27.63 ± 4.03	Ns
PhA	6.41 ± 1.18	6.18 ± 1.11	6.37 ± 0.83	Ns
Superior segment				
Rz	516.4 ± 97.54	468.34 ± 41.34	492.83± 83.89	Ns
Xc	52.02 ± 9.49	52.27 ± 10.14	53.11 ± 4.99	Ns
PhA	5.7 ± 0.67	6.35 ± 1.57	6.23 ± 1.06	Ns
Inferior segment				
Rz	520.08 ± 26.15	437.3 ± 75.87	491.45± 50.91	Ns
Xc	53.3 ± 19.45	46.81 ± 15.56	54.8 ± 7.67	Ns
PhA	6.39 ± 1.21	6.25 ± 1.13	6.34 ± 0.75	Ns
Right thorax				
Rz	19.63 ± 3.43	19 ± 5.32	18.97 ± 2.59	Ns
Xc	11.45 ± 1.94	12.83 ± 3.33	10.58 ± 0.57	Ns
PhA	30.48 ± 5.09	30.81 ± 16.99	29.47 ± 3.51	Ns
Left thorax				
Rz	19.44 ± 3.79	21.58 ± 4.03	19.1 ± 2.69	Ns
Xc	11.27 ± 1.89	11.33 ± 1.04	10.58 ± 0.33	Ns
PhA	30.52 ± 6.53	28.19 ± 5.31	27.57 ± 7.72	Ns

Caption: PhA: phase angle; Rz: resistance; Xc: reactance. NS: not significant.

## Data Availability

Original data are collected and protected in a local database under the surveillance of LS.

## Abbreviations

BMI	body mass index
IPAQ	International Physical Activity Level Questionnaire
MEDI-LITE	Mediterranean Diet Adhesion Questionnaire
Peak VO2 %	percentage of maximum oxygen consumption on peak predicted value for the subjects
VO2/HR %	percentage of peak oxygen pulse predicted value for the subjects
VE/VCO2	slope between ventilation and carbon dioxide production
PhA	phase angle
FFM	lean mass
FM	fat mass
ASMM	appendicular lean mass
J FFM	appendicular mass calculated with Jansenn formula
Rz	resistance
Xc	reactance
BM	basal metabolism
BCM	body cell mass
ECW	extracellular water
ICW	intracellular water
TBW	total body water
